# Zoledronic acid induces ferroptosis by reducing ubiquinone and promoting HMOX1 expression in osteosarcoma cells

**DOI:** 10.3389/fphar.2022.1071946

**Published:** 2023-01-04

**Authors:** Tianhao Ren, Ju Huang, Wei Sun, Guangze Wang, Yuwen Wu, Zewei Jiang, Yingshuai Lv, Guang Wu, Jiawei Cao, Min Liu, Haihua Gu

**Affiliations:** ^1^ Key Laboratory of Laboratory Medicine, Ministry of Education, Wenzhou Key Laboratory of Cancer Pathogenesis and Translation, School of Laboratory Medicine and Life Sciences, Wenzhou Medical University, Wenzhou, China; ^2^ Department of Orthopedics, Third Affiliated Hospital of Wenzhou Medical University, Wenzhou, China

**Keywords:** zoledronic acid, ferroptosis, ubiquinone, HMOX1, osteosarcoma

## Abstract

**Aims:** Ferroptosis plays important roles in tumorigenesis and cancer therapy. Zoledronic acid is known to inhibit the activity of farnesyl pyrophosphate synthase, a key enzyme in the mevalonate pathway. We examined whether zoledronic acid can inhibit the growth of osteosarcoma cells by inducing ferroptosis.

**Methods**: Cell viability was analyzed by using CCK8 reagent and counting cells with trypan blue exclusion. Ferroptosis markers including lipid peroxide and *PTGS2* expression were examined by flow cytometry, western blot, and quantitative PCR analyses. Cellular ubiquinone content was determined using high performance liquid chromatography. Ferrostatin-1 and RSL3 were used as the ferroptosis inhibitor and inducer respectively.

**Results**: Zoledronic acid treatment decreased cell viability and promoted the increase in lipid peroxide content and *PTGS2* expression. Addition of ferrostatin-1 reverted these effects of zoledronic acid on osteosarcoma cells, supporting a role of zoledronic acid in inducing ferroptosis. Mechanistically, zoledronic acid significantly decreased ubiquinone, a metabolite of the mevalonate pathway. Treating cells with exogenous ubiquinone prevented zoledronic acid-induced ferroptosis and decrease in the growth of osteosarcoma cells. In addition, zoledronic acid enhanced the expression of HMOX1, whereas knockdown of HMOX1 inhibited the zoledronic acid-induced increase in lipid peroxide level and decrease in cell growth. Finally, zoledronic acid together with RSL3 significantly enhanced the inhibitory effect on the growth of osteosarcoma cells.

**Conclusion:** Our results indicate that zoledronic acid induces ferroptosis by decreasing ubiquinone content and promoting HMOX1 expression in osteosarcoma cells. Zoledronic acid together with ferroptosis inducer may be a promising new strategy for the treatment of osteosarcoma.

## Introduction

Osteosarcoma (OS), a bone malignancy that originates from primitive mesenchymal cells, is the most common primary bone cancer in children and adolescents (Gill and Gorlick, 2021). Metaphysis of the long bones is the most frequent site of origin for osteosarcoma in patients (Sadykova et al., 2020). The standard treatment for osteosarcoma includes surgery and chemotherapy, before and after surgical resection (Benjamin, 2020). However, chemotherapy resistance is common with the increased use of chemotherapeutic drugs (Chou and Gorlick, 2006). Metastasis and chemotherapy resistance are two major factors affecting the survival of patients with osteosarcoma (Lu et al., 2020). Therefore, finding new therapeutic targets and strategies should help improve the long-term survival of patients with osteosarcoma.

Ferroptosis is an iron-dependent regulated cell death, resulting from rupture of the plasma membrane caused by high levels of lipid peroxides formed through oxidation of polyunsaturated fatty acid by reactive oxygen species (lipid ROS) (Stockwell et al., 2017). Ferroptosis can be regulated by exogenous and endogenous pathways. The exogenous pathways are initiated by inhibiting cell membrane transporters such as cystine/glutamate transporters (also known as the Xc^−^ system) or by activating iron transporters (i.e., transferrin and lactotransferrin). The endogenous pathways are activated through suppression of the activity of intracellular antioxidant enzymes including glutathione peroxidase 4 (GPX4). Lipid ROS is accumulated due to the decreases in intracellular glutathione (GSH) level and GPX4 activity, promoting ferroptosis (Ursini and Maiorino, 2020). In addition, other non-classical pathways have been implicated in regulating ferroptosis. Ferroptosis inhibitor protein 1 (FSP1) can convert intracellular ubiquinone (CoQ10) to the reduced form of panthenol, which blocks lipid peroxidation and ferroptosis (Doll et al., 2019). HMOX1, a heme oxygenase, can breakdown heme and release Fe^2+^ at the same time, resulting in the elevated intracellular concentration of Fe^2+^ and induction of ferroptosis (Chang et al., 2018).

In recent years, many studies have begun to reveal that inducing ferroptosis can be explored to treat cancer. Since many current cancer regimens work by inducing apoptosis, once escaping from apoptosis, tumor cells can develop drug resistance (Liang et al., 2019). Therefore, induction of ferroptosis is an attractive alternative strategy for cancer therapy. Traditional ferroptosis inducers RSL3 and Erastin have been shown to have significant antitumor effects in some musculoskeletal cancer, indicating the promise of targeting ferroptosis in the treatment of this type of cancer (Codenotti et al., 2018; Brack et al., 2020; Dächert et al., 2020). However, few studies have explored the application of inducing ferroptosis in treating osteosarcoma (Luo et al., 2021).

Zoledronic acid is a new generation of bisphosphonates, which has shown broader application prospects than the previous generation of bisphosphonates. It is well tolerated in the treatment of osteoporosis (Black et al., 2007). The US Food and Drug Safety Administration also approved zoledronic acid in combination with systemic therapy for the treatment of patients with bone metastases from solid tumors because it has been shown to be effective against many types of solid tumors (Santini et al., 2003; Rosen et al., 2004; Hatoum et al., 2008). Published studies have shown that zoledronic acid has therapeutic effects on tumors, including osteosarcoma by inhibiting cell proliferation and motility and inducing apoptosis and autophagy (Kubista et al., 2006; Dass and Choong, 2007; Oh et al., 2019; Kameda et al., 2021; Liu et al., 2021). However, it is not clear whether zoledronic acid can inhibit the growth of cancer cells including osteosarcoma cells through inducing ferroptosis. Interestingly, zoledronic acid has been reported to bind and inhibit the activity of farnesyl pyrophosphate synthase (FPPS), a key enzyme in the mevalonate pathway (Reszka and Rodan, 2004; Tang et al., 2017). Besides cholesterol, CoQ10, also a downstream lipid product in the mevalonate pathway (Thelin et al., 1994), is an important regulatory molecule that can inhibit ferroptosis in cells (Doll et al., 2019). In this study, we examined whether zoledronic acid can inhibit the growth of osteosarcoma cells by inducing ferroptosis.

## Materials and methods

### Cell culture and reagents

293T17 and Osteosarcoma cell lines MG63, U2-OS, HOS-MNNG were from National Collection of Authenticated Cell Cultures (Shanghai, China). Cells were cultured in Dulbecco’s Modified Eagle’s Medium (Gibco, California, United States) supplemented with 10% fetal bovine serum (Lonsera, Shanghai, China), 100 units/ml penicillin and 100 μg/ml streptomycin (Beyotime Institute of Biotechnology, Jiangsu, China) in a 37°C incubator with 7.5% CO_2_. Zoledronic acid (4 mg/5 ml) was from Novartis (Basel, Switzerland). CoQ10 (cat# 347174-05-4), ferrostatin-1 (cat# 303-98-0), and RSL3 (cat# 1219810-16-8) were purchased from MedChemExpress (Monmouth Junction, United States).

### Construction of shRNA expression vectors

For HMOX1 knockdown, two different HMOX1 shRNAs (sh#1 and sh#5) were inserted into the PLKO.1-puro plasmid. The two HMOX1 shRNA sequences are: sh#1: ACA​GTT​GCT​GTA​GGG​CTT​TAT; sh#5: GCT​GAG​TTC​ATG​AGG​AAC​TTT. PLKO.1-control-shRNA plasmid was used as described (Fang et al., 2019).

### Lentivirus production and infection

PLKO.1 lentiviruses were produced in 293T17 cells as described (He et al., 2017). Osteosarcoma cells were incubated with lentiviral supernatants and 8 μg/ml of polybrene (Sigma, St. Louis, United States) for 16 h. Twenty-six hours post-infection, stable pools of cells were selected with 1.3 μg/ml puromycin (Invitrogen, San Diego, United States) for 3 days before being used for subsequent experiments.

### Cell growth assay

Viable cells were analyzed by Cell Counting Kit-8 (CCK8) (DOJINDO, Japan) according to the manufacturer’s protocols. Cells were seeded at a density of 2 × 10^3^/well in 100 μL of medium into 96-well plates (Corning, New York, United States), and treated with various concentrations of zoledronic acid, ferrostatin-1, and CoQ10 alone or together for 72 h. Ferrostatin-1 was used at 5 μM to inhibit ferroptosis according to a published study (Dächert et al., 2020). CoQ10 was used at 0.75 μM because CoQ10 at 5 μM reduced the growth of osteosarcoma cell lines used in this study in a 3-day assay. Subsequently, CCK-8 reagent (10 μL) was added to each well, incubated for 2 h, and analyzed at 450 nm using a Fluoroskan microplate reader (Thermo Fisher, Waltham, MA, United States). The relative cell growth was presented as the absorbance.

### Quantitative (q)-PCR for mRNA level

Total RNA was extracted from osteosarcoma cells using Trizol Reagent (Invitrogen, California, United States) according to the manufacturer’s instruction. First strand cDNA synthesis and amplification were performed using PrimeScript™ RT reagent Kit with gDNA Eraser (TAKARA, Shiga, Japan). q-PCR was used to quantify the expression of PTGS2 and HMOX1 mRNAs using SYBR^®^ Premix Ex TaqII (Vazyme, Nanjing, China). GAPDH was used as the internal control gene. Gene specific primers were purchased from Genewiz Biological Technology (Suzhou, China). The primer sequences for q-RCR were listed as below: PTGS2, forward 5′-TGT​GCA​ACA​CTT​GAG​TGG​CT-3′, reverse 5′-ACT​TTC​TGT​ACT​GCG​GGT​G-3’. HMOX1, forward 5′-GAA​GAA​GGT​GCT​AAT​GTC​CTG​AC-3′, reverse 5′-GTC​CCA​GAC​TGT​AAT​TTC​GCC-3’. GAPDH, forward 5′-AGA​TCC​CTC​CAA​AAT​CAA​GTG​G-3′, reverse 5′-GGC​AGA​GAT​GAT​GAC​CCT​TTT-3’.

### Western blot

Western blot analysis was performed as described (Fang et al., 2019). Cell lysate from 20,000 cells from each sample lysed directly in 1XSDS sample buffer (50 mM Tris-HCl pH 6.8, 2% SDS, 10% glycerol, 2.5% β-mercaptoethanol, 12.5 mM EDTA, 0.02% bromophenol blue) was loaded in each lane of SDS-PAGE. Antibodies recognizing PTGS2 (cat# 12282, 1:2000 dilution) and β-actin (cat# 3700, 1:5000 dilution) were purchased from Cell Signaling Technology (Boston, United States). Antibodies recognizing HMOX1 (Cat# 10701-1-AP, 1:2000 dilution) were from Proteintech (Wuhan China). HRP- conjugated secondary antibodies (A0216 and A0208, 1:3000 dilution) were from Beyotime Institute of Biotechnology.

### Extraction of CoQ10 from osteosarcoma cells

Osteosarcoma cells were plated in a 15-cm dish at about 50% confluency and incubated with 20 μM zoledronic acid for 24 h. Cells were subsequently harvested with trypsin digestion, pelleted at 1000 rpm, resuspended with ethanol, and broken by ultrasound at 500 W for 20 min. Lysates were incubated at room temperature for 30 min before being centrifuged at 14000 rpm for 5 min to collect supernatants.

### Quantification of cellular CoQ10 using HPLC-UV

Cellular CoQ10 level was determined by HPLC-UV as described (Nierobisz et al., 2010; Rácz et al., 2015) with some modifications. Briefly, HPLC-UV was performed by using Agilent 1260 (California, United States) with a C18 column (4.6 mm × 250 mm, 5 μm) (Thermo Fisher, California, United States). The column temperature was set at 40°C, the detection wavelength was 275 nm, the flow rate was 1 ml/min, the sample volume was 20 μL, and the mobile phase was 20% methanol and 80% ethanol. The CoQ10 standard solutions of 10, 1, 0.1, and 0.01 μM were used to generate the standard curve according to the quantity of CoQ10 and the response peak area of HPLC. Subsequently, CoQ10 contents in samples were analyzed, quantified, and presented as pmole of CoQ10 per million cells.

### Analysis of lipid peroxidation using flow cytometry

Osteosarcoma cells were seeded in 12-well plates, and incubated with zoledronic acid (20 μM), CoQ10 (5 μM), and RSL3 (50/100 nM) alone or together for 24 h. The concentrations of CoQ10 and RSL3 used in this study were chosen according to published studies (López et al., 2010) (Zilka et al., 2017). For the final 30 min, cells were incubated with 5 μM BODIPY-C11 (Thermo Fisher), then were digested with trypsin, pelleted and washed with PBS before being analyzed by flow cytometry (BD Accuri C6). In each experiment, the gating strategy for analysis was determined as below. First, cells that were not cell debris and aggregates from the vehicle control group were selected. Then, in this selected population of cells, fluorescent level from cells with top 5% of the highest fluorescent levels was set as the basal lipid-ROS level. This exact gating was applied to other treatment samples to analyze different lipid ROS level for different treatment sample in the same experiment.

### Statistical analysis

Statistical analyses were performed using GraphPad Prism, version 8.0. Student t-test was used to compare data between two groups. One-way ANOVA with Bonferroni’s multiple comparison test correction was used to analyze data among multiple groups. Two-way ANOVA was used to analyze differences with two independent factors. All statistical tests were two-sided, and *p* < 0.05 was considered statistically significant. Results shown are representative from three independent experiments with similar results. For the analysis of all the quantitative data, triplicate data points for each sample were used to calculate the mean with SEM in each independent experiment.

## Results

### Zoledronic acid inhibits the growth of osteosarcoma cells

**FIGURE 1:**
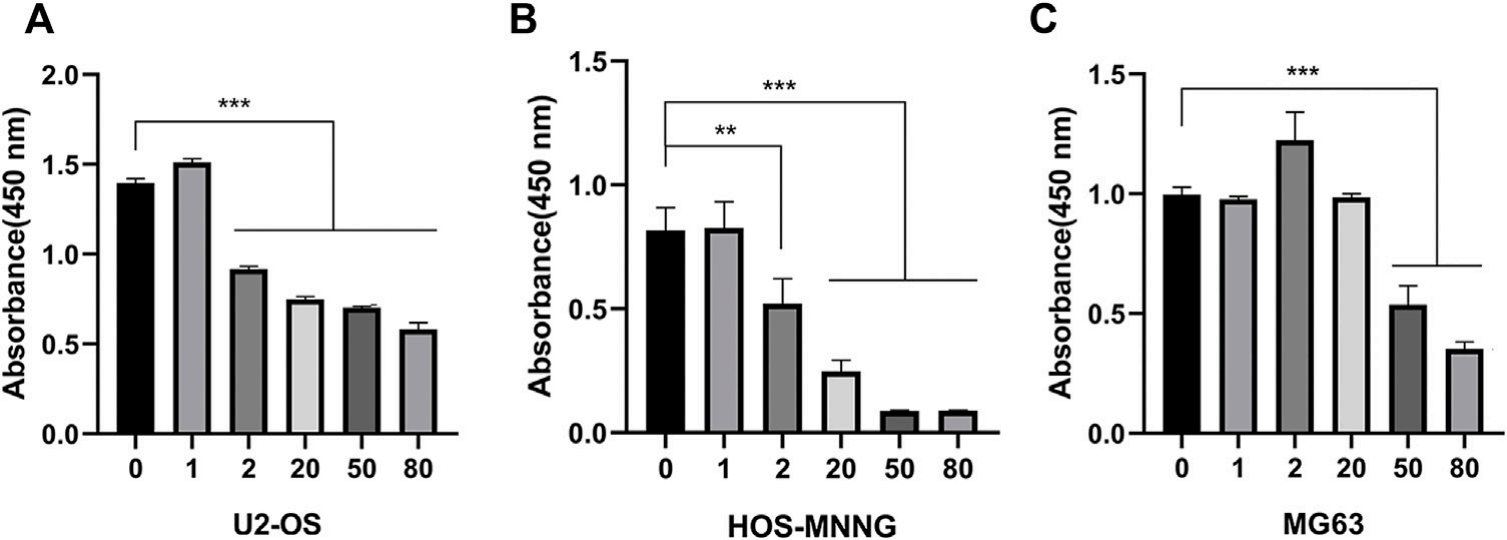
Zoledronic acid inhibits the growth of osteosarcoma cells. U2-OS **(A)**, HOS-MNNG **(B)** and MG63 **(C)** were treated with 0, 1, 2, 20, 50, 80 μM zoledronic acid for 3 days, respectively. The viable cells were detected by CCK8 reagent. The means ± SEM are indicated, *n* = 3. **p < 0.01, ***p < 0.001.

We examined the effect of zoledronic acid on the growth of different osteosarcoma cell lines by treating existing osteosarcoma cell lines U2-OS, HOS-MNNG, and MG63 with different concentrations of zoledronic acid (ZA) and examining cell viability. Zoledronic acid inhibited the growth of the three cell lines in a concentration-dependent manner. HOS-MNNG was more sensitive to ZA, whereas MG63 was less sensitive to ZA treatment. The IC50 of ZA for HOS-MNNG, U2-OS, and MG63 were 4.2, 13.1, and 49.1 μM, respectively, which were calculated based on the dose response curves (Figures 1A–C).

### Zoledronic acid induces ferroptosis in osteosarcoma cells

**FIGURE 2:**
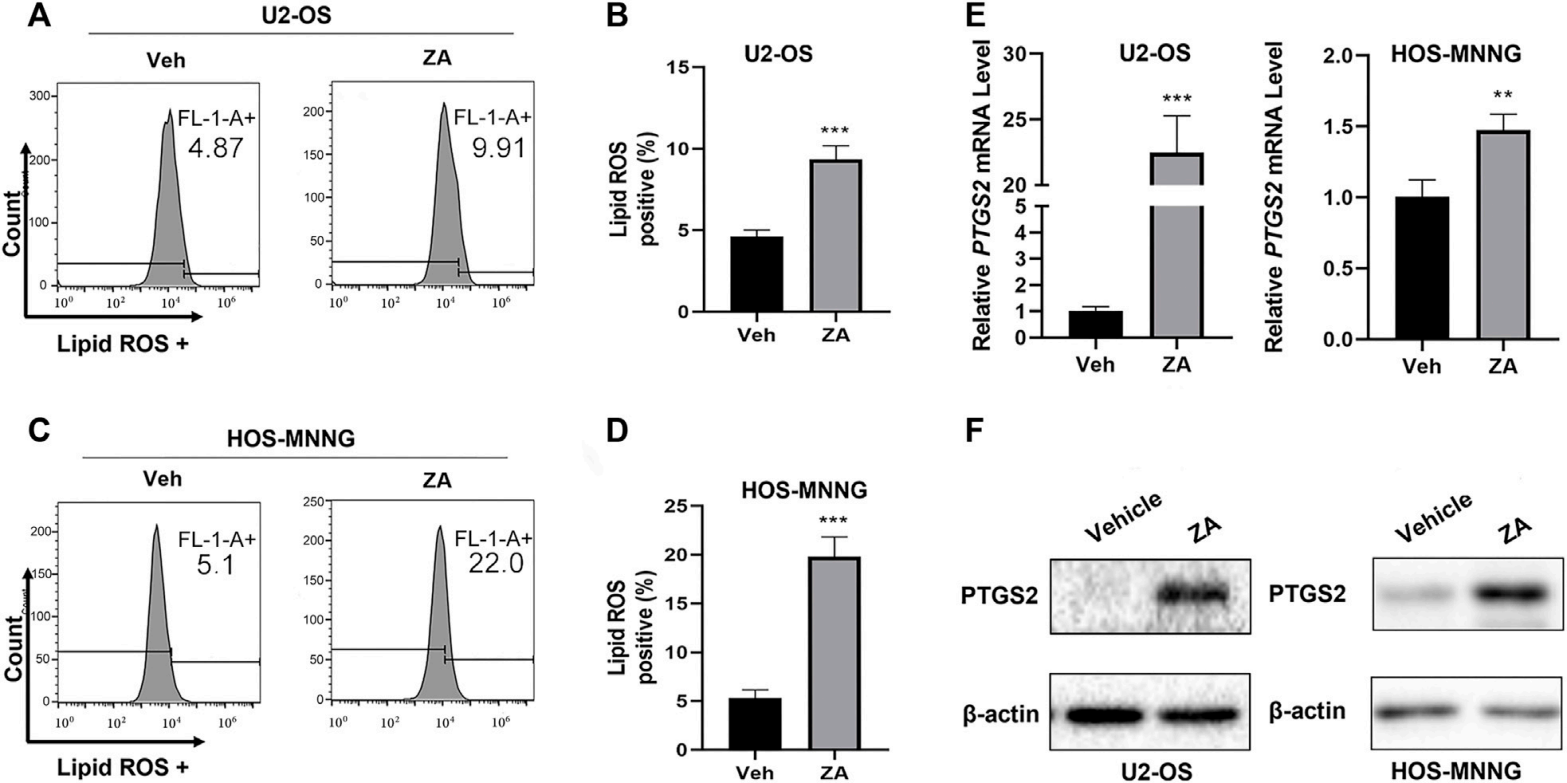
Zoledronic acid induces ferroptosis in osteosarcoma cells. **(A–D)** Zoledronic acid (ZA) increases lipid peroxide levels in osteosarcoma cells. U2-OS **(A,B)** and HOS-MNNG **(C,D)** cells were treated with 20 μM ZA for 24 h and incubated with BODIPY 581/591C11 (5 μM) dye for 30 min. Flow cytometry was used to detect Lipid ROS levels. **(B,D)** Quantitation of the flow cytometry plots in triplicates for **(A)** and **(C)** are shown. ZA treatment enhances the mRNA **(E)** and protein **(F)** levels of PTGS2. mRNA and protein were extracted from ZA (20 μM) treated (24 h) cells, respectively. PTGS2 mRNA level was analyzed by qPCR, and PTGS2 protein level was examined by western blot. The means ± SEM are indicated, *n* = 3. *p < 0.05, **p < 0.01, ***p < 0.001.

To test whether zoledronic acid induces ferroptosis in osteosarcoma cells, we examined the levels of ferroptosis markers in osteosarcoma cells. An important hallmark of ferroptosis is the accumulation of oxidized Lipid ROS. Zoledronic acid treated U2-OS and HOS-MNNG cells were analyzed by flow cytometry to detect the lipid ROS levels using the lipid peroxide sensing reagent (BODIPY 581/591 C11). We found that zoledronic acid promoted the accumulation of lipid ROS in osteosarcoma cells compared with vehicle control (Figures 2A–D). Ferroptosis is also frequently accompanied by the increased expression of ferroptosis-specific genes including *PTGS2*. Quantitative PCR and immunoblotting analyses revealed that zoledronic acid significantly enhanced *PTGS2* mRNA (Figure 2E) and protein (Figure 2F) levels. These data show that zoledronic acid induces ferroptosis in osteosarcoma cells. We did not examine the effect of zoledronic acid in inducing ferroptosis in MG-63 cells in detail in the rest of our study since MG63 was less sensitive to zoledronic acid treatment (Figure 1C).

### Zoledronic acid reduces the survival of osteosarcoma cells through ferroptosis

**FIGURE 3:**
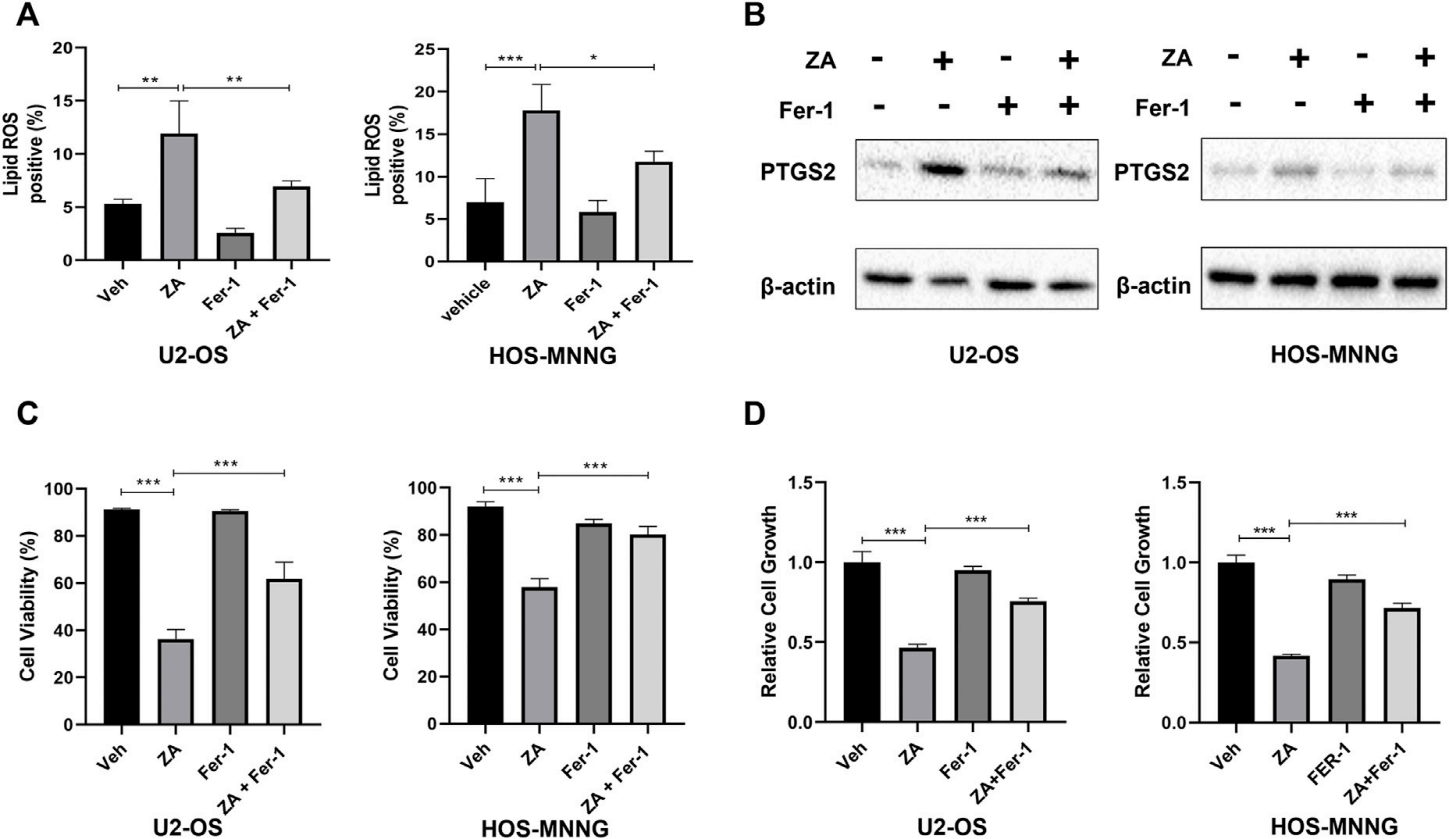
Zoledronic acid decreases cell survival of osteosarcoma cells through ferroptosis. **(A)** Ferrostain-1 reduces ZA-enhanced lipid ROS level. U2-OS and HOS-MNNG cells were treated with vehicle (Veh), ZA (20 μM) and Ferrostatin-1 (Fer-1) (5 μM) alone or together for 24 h. Lipid ROS were detected by flow cytometry. **(B)** Ferrostain-1 reduces ZA-enhanced PTGS2 protein level. Cells were treated with ZA (20 μM) and Fer-1 (5 μM) alone or together respectively for 24 h and immunoblotted using PTGS2 antibody. **(C,D)** Ferrostain-1 prevents decreased cell survival induced ZA treatment. Cells were treated with ZA (20 μM) and Fer-1 (5 μM) either alone or together for 72 h, respectively. Viable and total cells were counted by trypan blue exclusion, respectively **(C)**. The percent of viable cells was calculated. In addition, viable cells were assayed using CCK8 reagent **(D)**. The means ± SEM are indicated, *n* = 3. *p < 0.05, **p < 0.01, ***p < 0.001.

To further support that zoledronic acid induces ferroptosis, we tested the effects of ferrostatin-1, a ferroptosis inhibitor, on zoledronic acid-induced ferroptosis markers. Ferrostatin-1 is a novel synthetic antioxidant that prevents polyunsaturated fatty acids from undergoing oxidation in cell membrane (Zilka et al., 2017), thereby inhibiting ferroptosis. The addition of ferrostatin-1 was found to partially suppress the increase in lipid peroxide levels induced by zoledronic acid (Figure 3A). In addition, Ferrostatin-1 treatment prevented the increase in PTGS2 protein level induced by zoledronic acid (Figure 3B). The above data revealed that zoledronic acid indeed induces ferroptosis in osteosarcoma cells.

Next, we explored whether ferrostatin-1 can hinder zoledronic acid-induced decrease in the survival of osteosarcoma cells. Viable U2-OS and HOS-MNNG cells, treated with zoledronic acid and ferrostatin-1 either separately or together, were counted by trypan blue exclusion and analyzed using CCK8 reagent. Zoledronic acid treatment reduced cell survival, whereas the addition of ferrostatin-1 partially reversed the zoledronic acid-induced decrease in cell survival (Figure 3C) and viability (Figure 3D). These results indicate that zoledronic acid decreases the survival, and therefore the growth of osteosarcoma cells by inducing ferroptosis.

### CoQ10 inhibits zoledronic acid-induced ferroptosis in osteosarcoma cells

**FIGURE 4:**
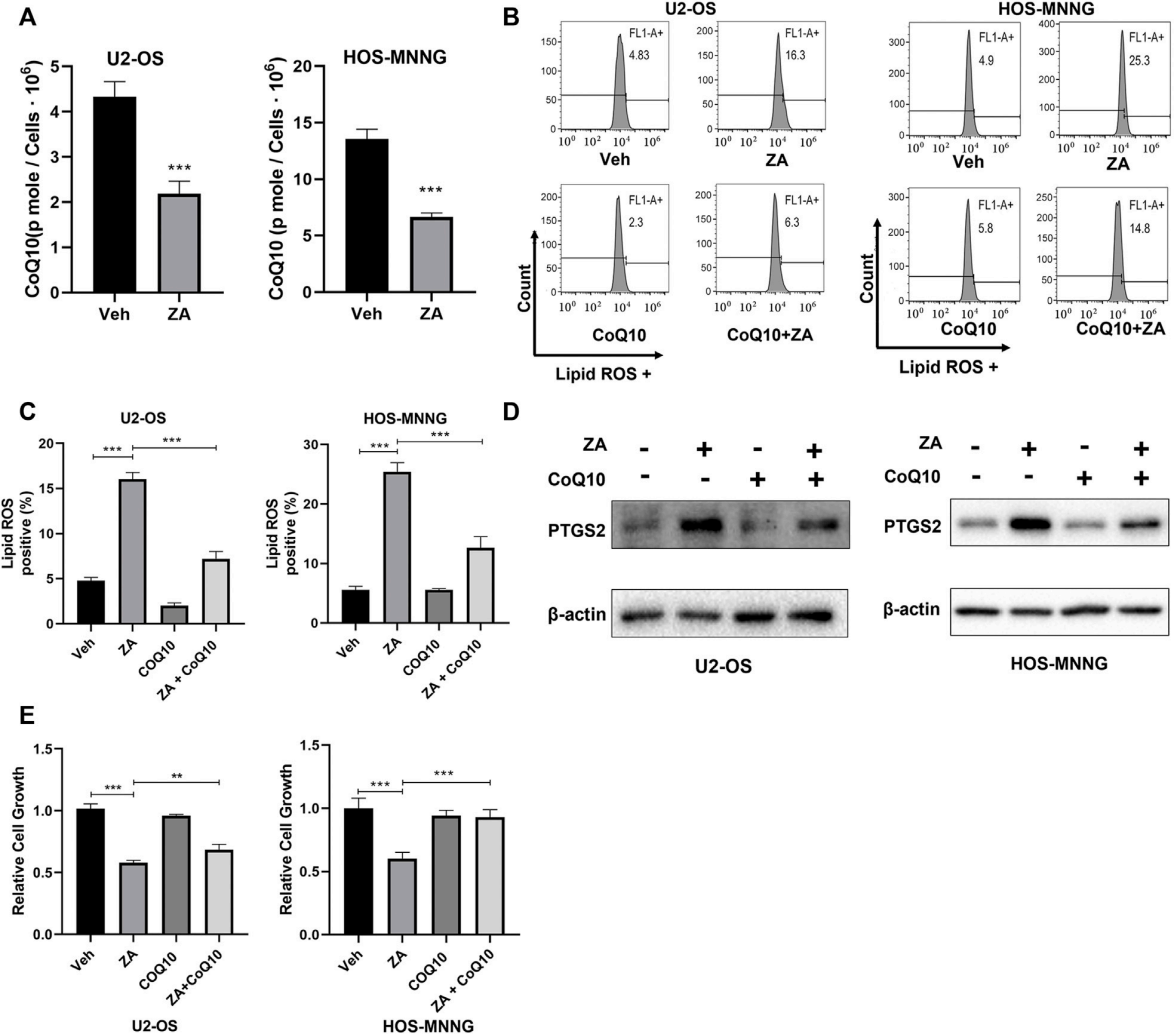
Zoledronic acid induces ferroptosis and inhibits cell growth in part through reducing CoQ10 content in osteosarcoma cells. **(A)** ZA treatment reduces CoQ10 level in osteosarcoma cells. U2-OS and HOS-MNNG cells were treated with vehicle (Veh) and ZA (20 μM) for 24 h, and broken by ultrasound. CoQ10 was extracted by ethanol and was quantified by HPLC. **(B,C)** Addition of exogenous CoQ10 inhibits ZA-enhanced lipid ROS level. Cells were treated with Veh, ZA (20 μM) and CoQ10 (5 μM) alone or together for 24 h and lipid ROS level was analyzed. **(C)** Quantitation and statistical analysis of the data shown in **(B)**. **(D)** Addition of exogenous CoQ10 decreases ZA-enhanced PTGS2 protein level. Cells were treated with Veh, ZA (20 μM) and CoQ10 (5 μM) alone or together for 24 h. Western blot was used to detect PTGS2 protein expression. **(E)** Exogenous CoQ10 prevents ZA-induced decrease in cell growth. Cells were treated with Veh, ZA (20 μM), or CoQ10 (0.75 μM) alone or together for 72 h. Then, the cell growth was examined by CCK8 reagent and analyzed. The means ± SEM are indicated, *n* = 3. *p < 0.05, **p < 0.01, ***p < 0.001.

Zoledronic acid can inhibit the activity of FPPS, a key enzyme for the generation of the upstream metabolite for CoQ10 biosynthesis. Therefore, we tested whether zoledronic acid induces ferroptosis by decreasing the cellular content of CoQ10. First, HPLC was used to analyze the levels of CoQ10 in osteosarcoma cells treated with zoledronic acid. Zoledronic acid treatment significantly reduced CoQ10 contents compared with vehicle control in U2-OS and HOS-MNNG cells (Figure 4A). Next, we examined the effects of exogenous CoQ10 on zoledronic acid-induced ferroptosis and decrease in cell growth. U2-OS and HOS-MNNG cells were treated with zoledronic acid and exogenous CoQ10 either separately or together. Zoledronic acid treatment increased Lipid ROS levels, whereas the addition of exogenous CoQ10 significantly inhibited the increase of Lipid ROS levels induced by zoledronic acid in U2-OS and HOS-MNNG cells (Figures 4B,C). In addition, zoledronic acid treatment significantly increased PTGS2 protein levels, whereas adding exogenous CoQ10 blocked the zoledronic acid-induced increase in PTGS2 levels in osteosarcoma cells (Figure 4D). Finally, we investigated the effect of exogenous CoQ10 on the inhibition of cell growth by zoledronic acid. Zoledronic acid again significantly inhibited the growth of osteosarcoma cells, whereas adding exogenous CoQ10 rescued the zoledronic acid-induced growth inhibition in U2-OS and partially in HOS-MNNG (Figure 4E). These results indicate that zoledronic acid promotes ferroptosis and inhibits cell growth in osteosarcoma cells, at least by decreasing intracellular CoQ10 levels.

### HMOX1 mediates zoledronic acid-induced ferroptosis in osteosarcoma cells

**FIGURE 5:**
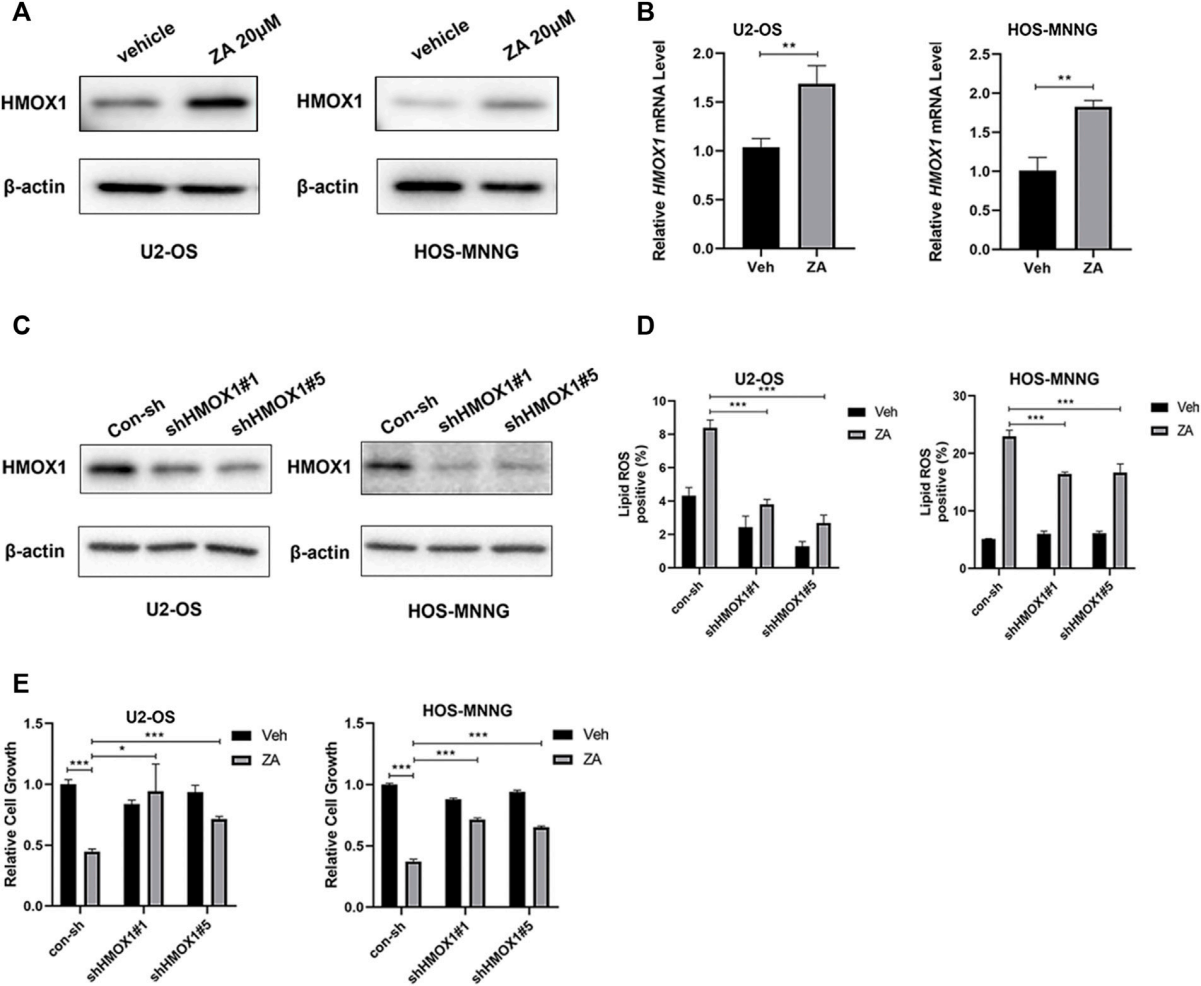
HMOX1 mediates zoledronic acid-induced ferroptosis in osteosarcoma cells **(A,B)** ZA treatment increases HMOX1 expression. U2-OS and HOS-MNNG cells were treated with vehicle (Veh) and 20 μM ZA for 24 h. HMOX1 protein levels were detected by western blot analysis **(A)** and HMOX1 mRNA levels were quantified by qPCR **(B)**. **(C)** HMOX1 protein level was efficiently knocked down by two different shRNAs in U2-OS and HOS-MNNG cells. **(D)** Knockdown of HMOX1 decreases ZA-induced increase in lipid ROS level. Indicated cells expressing control-shRNA (con-sh) and two different HMOX1 shRNAs (shHMOX#1 and shHMOX#5) were treated with Veh and 20 μM ZA for 24 h, and lipid ROS levels were analyzed. **(E)** Knockdown of HMOX1 prevents ZA-induced decrease in cell growth. Indicated cells expressing con-sh, shHMOX#1 and shHMOX#5 were treated with veh and ZA (20 μM for U2OS, 40 μM for HOS-MNNG) for 72 h. Cell growth was examined by CCK8 reagent and analyzed. The means ± SEM are indicated, *n* = 3. *p < 0.05, **p < 0.01, ***p < 0.001.

HMOX1 is a heme oxygenase that acts in a non-classical pathway to enhance ferroptosis (Chang et al., 2018). We examined whether zoledronic acid treatment also induces ferroptosis by increasing HMOX1 expression. Zoledronic acid treatment significantly enhanced HMOX1 protein and mRNA levels in osteosarcoma cells (Figures 5A,B). To further test whether the increased HMOX1 level mediates zoledronic acid-induced ferroptosis, two different shRNAs were used to knock down HMOX1 protein expression in U2-OS and HOS-MNNG cells (Figure 5C). Zoledronic acid significantly increased lipid ROS level in control cells, whereas the ability of zoledronic acid to increase lipid ROS level was blocked in HMOX1 knockdown U2-OS cells (Figure 5D left) and partially blocked in HMOX1 knockdown HOS-MNNG cells (Figure 5D right). In addition, HMOX1 knockdown prevented zoledronic acid-induced decrease in cell growth in U2-OS and HOS-MNNG cells (Figure 5E). These results indicate that zoledronic acid also promotes ferroptosis by increasing HMOX1 expression in osteosarcoma cells.

### Ferroptosis inducer RSL3 enhances zoledronic acid-induced ferroptosis and inhibition of cell growth in osteosarcoma cells

**FIGURE 6:**
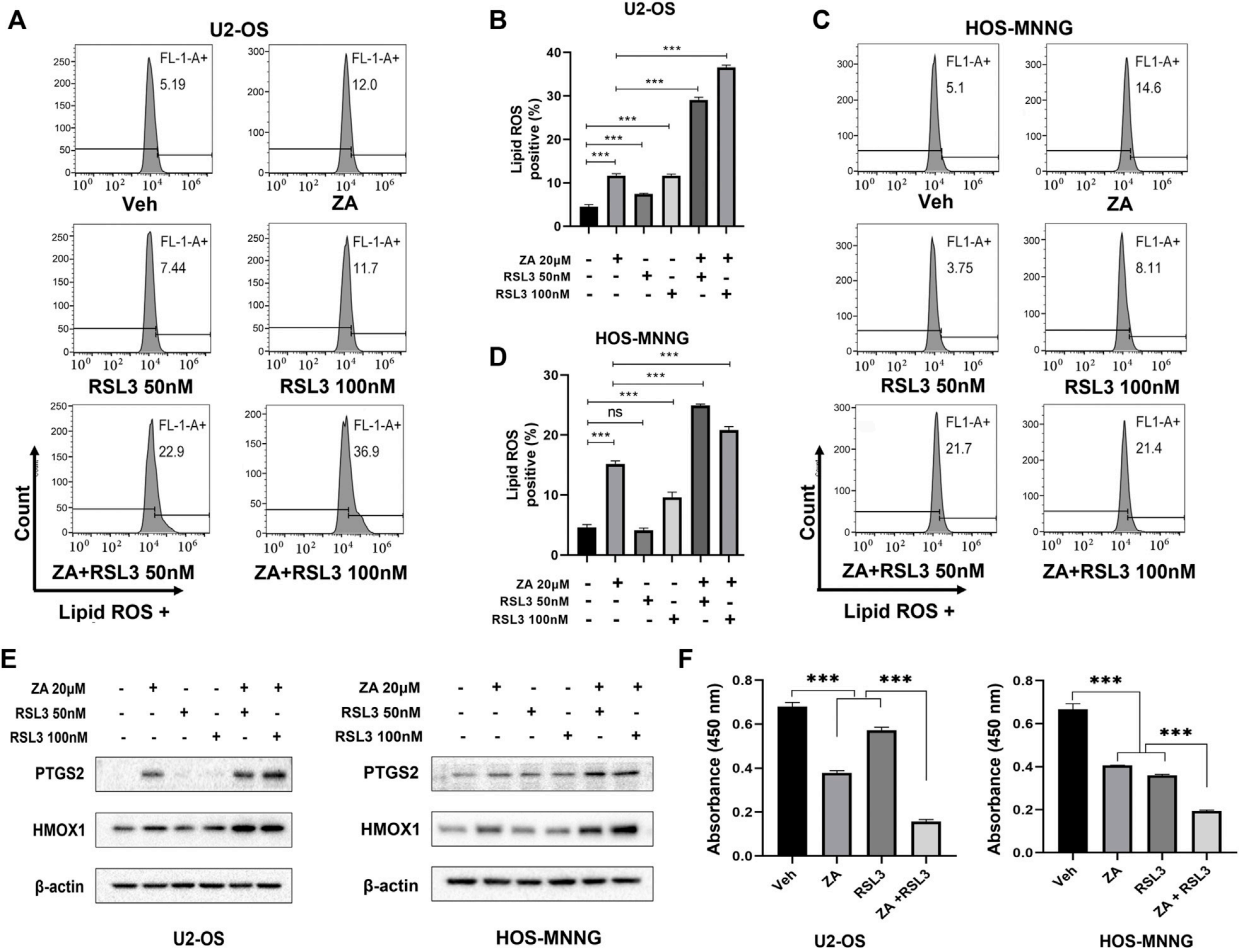
Ferroptosis inducer enhances zoledronic acid-induced ferroptosis and inhibition of cell growth in osteosarcoma cells. U2-OS **(A, B)** and HOS-MNNG **(C,D)** cells were treated with vehicle (Veh), ZA (20 μM), or indicated concentrations of Ferroptosis inducer RSL3 alone or together for 24 h, and lipid ROS levels were analyzed **(A,C)**. **(B,D)** Quantitation of the flow cytometry data shown in **(A,C)**, respectively. **(E)** Cells were treated with zoledronic acid (20 μM), RSL3 (50 nM, or 100 nM) alone or together for 24 h, and the protein levels of PTGS2 and HMOX1 were examined by western blot analysis. **(F)** RSL3 enhances ZA-induced inhibition of cell growth. Cells were treated with ZA (20 μM) and RSL3 (100 nM) alone or together for 72 h before being analyzed by CCK8 reagent. The means ± SEM are indicated, *n* = 3. *p < 0.05, **p < 0.01, ***p < 0.001.

RSL3 induces ferroptosis by binding and inactivating GPX4 (Yang et al., 2014). We hypothesized that zoledronic acid and RSL3 may enhance ferroptosis, further inhibiting the growth of osteosarcoma cells. To test this possibility, cells were treated with zoledronic acid and RSL3 either alone or in combination. Zoledronic acid or RSL3 treatment increased the level of lipid peroxides compared with vehicle control, while zoledronic acid together with RSL3 further enhanced the level of lipid peroxides (Figures 6A–D). In addition, zoledronic acid with RSL3 significantly increased HMOX1 protein levels compared with either single agent treatment alone. Likewise, zoledronic acid together with RSL3 significantly further increased the protein levels of PTGS2 (Figure 6E) compared with zoledronic acid treatment alone. Furthermore, we examined the effect of zoledronic acid in combination with RSL3 on the growth of U2-OS and HOS-MNNG cells. The results revealed that zoledronic acid with RSL3 inhibited the growth of osteosarcoma cells more effectively than either single drug treatment (Figure 6F).

## Discussion

Our study illustrates for the first time that zoledronic acid can impair the growth of osteosarcoma cells by inducing ferroptosis. Zoledronic acid is a new generation of nitrogenous bisphosphonates and is currently a conventional first-line drug in the treatment of solid tumors with bone metastasis (Berenson, 2001). Zoledronic acid can directly inhibit cell proliferation (Peng et al., 2017) and induce apoptosis (Singireesu et al., 2018), as well as block migration and metastasis (Ory et al., 2005) of a variety of cancer cells including osteosarcoma cells. Zoledronic acid inducing ferroptosis in osteosarcoma cells are based on the following observations. Zoledronic acid enhanced lipid peroxide levels and PTGS2 expression, both of which are markers for ferroptosis (Figure 2). In addition, addition of ferroptosis inhibitor ferrostatin-1 blocked zoledronic acid-induced increases in lipid ROS level and PTGS2 expression (Figures 3A,B). Furthermore, ferrostatin-1 prevented zoledronic acid-induced decrease in cell survival in osteosarcoma cells (Figures 3C,D). Our data support that zoledronic acid inhibits the growth of osteosarcoma cells in part through ferroptosis.

Our data support a model that zoledronic acid causes ferroptosis likely through inhibition of FPPS and limiting the production of farnesyl pyrophosphate, consequently decreasing CoQ10 levels in osteosarcoma cells. Zoledronic acid has been shown to induce apoptosis in myeloma cells by decreasing geranylgeranyl pyrophosphate, a metabolite downstream of farnesyl pyrophosphate required for the geranylgeranylation of small GTPase proteins (Shipman et al., 1998). CoQ10, another downstream product of farnesyl pyrophosphate, is catalyzed by FSP1 into ubiquinol in cells, which can directly interfere the generation of lipid peroxides and suppress ferroptosis (Doll et al., 2019). Consistent with the inhibitory effect on FPPS, our result showed that zoledronic acid treatment reduced CoQ10 content (Figure 4A) and increased lipid ROS level (Figures 2A, B) in osteosarcoma cells. Similarly, a recent report shows that zoledronic acid can inhibit the CoQ10 biosynthesis in liver cells (Fernández-Del-Río et al., 2021). Importantly, we found that adding exogenous CoQ10 can suppress zoledronic acid-induced increased lipid ROS (Figures 4B,C) and the decreased cell growth (Figure 4F). These data support that zoledronic acid induces ferroptosis at least in part through the inhibition of FPPS and the reduction of CoQ10 content. However, we do not exclude that zoledronic acid may affect ferroptosis by changing the production of other metabolites in the mevalonate pathway.

Our result also indicates that zoledronic acid induces ferroptosis in osteosarcoma cells by promoting a rise in HMOX1 protein and mRNA level. HMOX1 has been reported to reduce heme to release Fe^2+^, leading to intracellular accumulation of labile iron Fe^2+^, which in turn increases lipid peroxide level and induces ferroptosis (Zheng and Conrad, 2020). A recent study shows that HMOX1 expression mediates ferroptosis induction by EF24, a synthetic analogue of curcumin, in osteosarcoma cells (Lin et al., 2021). It remains to be determined whether zoledronic acid and EF24 activate the same or different pathway to upregulate HMOX1 in osteosarcoma cells. *HMOX1* gene transcription can be subjected to complex regulation involving different transcription factors such as WT1, Sp1, C/EBPα, and Nrf2 (Wang et al., 2020; Zhang et al., 2021). Further studies are required to delineate which of these factors mediates zoledronic acid-induced increase in *HMOX1* mRNA expression in osteosarcoma cells.

Due to the lack of targeted therapy, osteosarcoma is commonly treated with standard chemotherapy, from which chemo-resistance is the recurring challenge (Liang et al., 2019). A variety of ferroptosis inducers including RSL3 have been reported to exhibit cytotoxicity against osteosarcoma cells (Liu and Wang, 2019), which illustrates activating ferroptosis as the promising therapy for the treatment of osteosarcoma. RSL3 induces ferroptosis by inhibiting the activity of GPX4. Since our study reveals that zoledronic acid promotes ferroptosis through decreasing cellular CoQ10 level, which is independent from the inhibition of GPX4 by RSL3, this explains the additive inhibitory effects of combining these two drugs on the growth of osteosarcoma cells. In the future, it will be interesting to explore whether zoledronic acid together with ferroptosis inducer can be used to overcome chemoresistance of osteosarcoma.

In summary, our study demonstrates for the first time that zoledronic acid inhibits the growth of osteosarcoma cells by inducing ferroptosis through reducing cellular CoQ10 content and upregulation of HMOX1 expression. Targeting ferroptosis by combining zoledronic acid with ferroptosis inducer may represent a promising new strategy to treat osteosarcoma.

## Data Availability

The original contributions presented in the study are included in the article/supplementary material, further inquiries can be directed to the corresponding authors.
